# State Estimation for Coupled Reaction-Diffusion PDE Systems Using Modulating Functions

**DOI:** 10.3390/s22135008

**Published:** 2022-07-02

**Authors:** David Pumaricra Rojas, Matti Noack, Johann Reger, Gustavo Pérez-Zúñiga

**Affiliations:** 1 Departamento de Ingeniería, Pontificia Universidad Católica del Perú, Avenida Universitaria 1801, Lima 15088, Peru; a20194219@pucp.edu.pe; 2 Control Engineering Group, Technische Universität Ilmenau, 98693 Ilmenau, Germany; matti.noack@tu-ilmenau.de (M.N.); johann.reger@tu-ilmenau.de (J.R.)

**Keywords:** state estimation, boundary observer, modulating function method, reaction-diffusion partial differential equations, coupled partial differential equations

## Abstract

Many systems with distributed dynamics are described by partial differential equations (PDEs). Coupled reaction-diffusion equations are a particular type of these systems. The measurement of the state over the entire spatial domain is usually required for their control. However, it is often impossible to obtain full state information with physical sensors only. For this problem, observers are developed to estimate the state based on boundary measurements. The method presented applies the so-called modulating function method, relying on an orthonormal function basis representation. Auxiliary systems are generated from the original system by applying modulating functions and formulating annihilation conditions. It is extended by a decoupling matrix step. The calculated kernels are utilized for modulating the input and output signals over a receding time window to obtain the coefficients for the basis expansion for the desired state estimation. The developed algorithm and its real-time functionality are verified via simulation of an example system related to the dynamics of chemical tubular reactors and compared to the conventional backstepping observer. The method achieves a successful state reconstruction of the system while mitigating white noise induced by the sensor. Ultimately, the modulating function approach represents a solution for the distributed state estimation problem without solving a PDE online.

## 1. Introduction

Many systems are modeled by partial differential equations (PDEs). Typical examples are solar collector systems [1], drilling systems [2,3], chemical reaction systems [4], medical imaging, seismic imaging, oil exploration, and computer tomography [5].

As stated in [6], state estimators are used to derive estimates of system variables that are difficult to measure directly and provide what is also called soft sensing of the state variable. Their recent applications extend across manufacturing and industrial processes.

A particular type of this kind of system are coupled reaction-diffusion PDEs. This type of system is characterized by a coupling between the system states in the PDEs. Every equation has the form of a reaction-diffusion PDE and is dependent on the other ones, making the system more complex in comparison to a normal PDE.

An example of such system is chemical tubular reactors [7] where the state variables, temperature and concentration, are coupled by the PDE related to each of these states.

For the control of this kind of system, a measurement of the whole spatial domain is often required and this requisite is nearly impossible with physical sensors. Because of this, observers are developed in order to estimate the whole state only with boundary measurements. An observer in combination with a control strategy can enable an output feedback control to achieve its goal with only boundary measurements that can be done with a sensor collocated at the boundary. Different kinds of observers, such as adaptive and iterative observers have been proposed in [8] and recursive observers-based methods have been introduced in [9]. Different other types of observers have been proposed for coupled dynamical cascade systems including ordinary differential equations (ODE) and PDEs, for example see [10]. An overall review of state estimation techniques in [11] shows the use of late lumping, optimal estimation, semi-group theory, the Lyapunov method, backstepping technique, and robust state estimation in real application scenarios.

One of the early attempts to the solution of the problem for coupled reaction-diffusion PDEs was done in [12]. The work was devoted to the solution of the stabilization problem with the same diffusivity parameters. In [13], using an approach similar to [12], the problem of state estimation for coupled reaction-diffusion PDEs with the same diffusivity parameters is addressed. The observer is designed in order to have a convergence in the estimation error system, transforming the original system through the use of the Kernel matrix. In [14] the extension is done for coupled reaction-advection-diffusion equations also with the same restriction of the same diffusivity parameters through the backstepping method. In [15] some of the results from [13] are used to design an observer for a coupled reaction-diffusion system with constant parameters that are used for an output feedback stabilization of this system.

Then, in [16,17] a next step in generalizing the type of systems approached is made by solving the stabilization and estimation for coupled reaction-diffusion systems with spatially varying reaction terms. In [17], the estimation is done for a 2-coupled reaction-diffusion PDE with spatially varying coefficients, which can be used to model the diffusion phenomena in lithium-ion batteries with electrodes that comprise multiple active materials. In [18], the approach is generalized to an n-coupled reaction-diffusion PDE with spatially varying coefficients.

The approach of the work here is based on the so-called modulating function (MF), introduced in the early 1950s by Shinbrot [19,20], to be used for parameter identification of ODEs. In this case, modulating functions are used for state estimation. This method reduces the original problem to a calculation of the coefficients that are used in the basis representation of the actual state through the solution of a linear system of equations, making the process of estimation much simpler to calculate and also less computationally intensive.

The modulating function-based method conceptionally differs from the PDE backstepping approach. It involves approximating the signals in a function basis representation, using specific properties of the modulating functions when applying a modulation operation to the original system, and transforming the original system into a series of algebraic relations. Often this process also involves some restrictions in the modulating functions that can be understood as an auxiliary system (also called signal modeling) to be resolved in a pre-processing before the estimation.

The method has been used for parameters and source estimation for one-dimensional first-order hyperbolic PDEs [21]. In this work, the modulating function method is used to estimate the source function and velocity for the wave equation. The work also explores the influence of different parameters in the method, such as the length of the basis, the size of the time window, and the type of basis functions chosen. The results also show the behavior of the estimation with respect to noise on the output of the system, featuring a good performance and robustness of the method. Ref. [22] developed fault detection and isolation for a parabolic PDE system using modulating functions, applying the method for a faulty heat conducting rod. This method traces back the fault detection problem to a trajectory planning problem using modulating functions obtained by the realization of a set-point change for their signal models and, using previous results on motion planning for distributed parameter systems, fault detection and isolation can be achieved.

The most recent result [23] is related to the state estimation for reaction-diffusion PDE with constant parameters. There the whole state is estimated from a measurement on the boundary. Through the use of modulating functions, the estimation problem is transformed into a linear system of equations for the coefficients of the basis expansion that represents the whole state. The resulting auxiliary system has a form very similar to the original system. This work constitutes the foundation for the present work since it demonstrates that the modulating function approach may successfully be used for reaction-diffusion PDE systems and how the state estimation can be achieved. In the present work, these results have been inspiring to formulate the problem for coupled reaction-diffusion PDE systems.

In this paper we provide an extension of the modulating function method to coupled reaction-diffusion PDE systems with spatially varying coefficients for state estimation. The method only requires the inputs of the system and a measurement of the state at one of the boundaries, achieving the objective of the soft sensor. This soft sensor provides the complete state information at any time and gives the representation of the state as a function basis expansion. The method provides an alternative solution to the problem of state estimation without need to calculate a PDE solution in real time. On the contrary, the modulating function-based method requires less computational cost enabling a real-time implementation for application.

The method presents advantages with respect to other observers such as the backstepping observers due to the efficient calculation of the state estimates, requiring only numerical integrations and matrix multiplications, whereas the backstepping observer requires an online calculation of a coupled PDE. The method also presents robustness against noise and is non-asymptotic by design, unlike asymptotic observers such as backstepping observers.

The paper is organized as follows: Section 2 presents the problem statement and some definitions that are useful in the solution of the problem. The use of modulating functions for the state estimation of an n-coupled reaction-diffusion PDE with spatially varying coefficients is proposed in Section 3. In Section 4, an implementation of the method developed in the last section is provided. Different parameters of the observer are tested in order to analyze their influence on the estimation, simulation results are presented and compared with other results. Finally, Section 5 holds our conclusions.

## 2. Estimation Problem Definition

The central subject of soft sensor design for coupled PDE systems involves a precise characterization of the considered PDE structure. In order to extract the state information from measured data, an adequate representation of the solution function in the form of a separable series expansion is established. It builds the foundation for extracting state information by means of the modulating function method by building a suitable input-output relation. The underlying modulation operator is defined with respect to time and space.

### 2.1. Coupled Reaction-Advection-Diffusion PDE

This work deals with the following class of linear systems modeled by *n*-coupled advection-diffusion-reaction equations with spatially varying coefficients:

(1) $$\left.\begin{array}{c} \frac{\partial W}{\partial t}\left(x,t\right)=\Sigma \left(x\right)\frac{\partial^{2}W}{\partial x^{2}}\left(x,t\right)+\Phi \left(x\right)\frac{\partial W}{\partial x}\left(x,t\right)+\Lambda \left(x\right)W\left(x,t\right) \end{array}\right.$$

where

$$W\left(x,t\right)={\left[\begin{array}{ccc} w_{1}\left(x,t\right) & \cdots & w_{n}\left(x,t\right) \end{array}\right]}^{⊤}$$

is the state vector and x∈Ω:=[0,L],t≥0. The coupling matrices Σ(x), Φ(x), $\Lambda \left(x\right)\in R^{n\times n}$ with corresponding component functions $\epsilon_{ij}\in C^{2}\left(\Omega \right),\phi_{ij}\in C^{1}\left(\Omega \right),\lambda_{ij}\in C^{0}\left(\Omega \right)$ for i,j=1,…,n, are the diffusion, advection, and reaction term coefficients, respectively. The system has the mixed-type boundary condition

$$P_{1}\frac{\partial W}{\partial x}\left(0,t\right)+P_{0}W\left(0,t\right)=F\left(t\right)$$

that can be a Dirichlet or Neumann-type boundary condition choosing either $P_{1}=0$ or $P_{0}=0$. Additionally, we have known actuation at the boundary, i.e.,

(2) $$Q_{1}\frac{\partial W}{\partial x}\left(L,t\right)+Q_{0}W\left(L,t\right)=U\left(t\right)$$

with inputs $U\left(t\right)\in R^{n}$ and measurements $Y\left(t\right)\in R^{n}$ as

$$Y\left(t\right)=R_{1}\frac{\partial W}{\partial x}\left(x^{*},t\right)+R_{0}W\left(x^{*},t\right)$$

at the boundary with $x^{*}=0$ or *L*. The task is to estimate the state W(x,t) with the measurements Y(t) and the inputs U(t) in the case of unknown initial conditions W(x,0).

### 2.2. Function Basis Expansion

Similar to the strategy in [23], a series expansion of the solution of system (1) is utilized for extracting the unknown state information. In the present work, it is assumed that the subsystem states $w_{l}\left(x,t\right),l\in \left\{1,\ldots ,n\right\}$, can be represented as

(3) $$w_{l}\left(x,t\right)=\sum_{k=0}^{\infty }c_{l}^{k}\left(t\right)\psi_{k}\left(x\right)$$

with $\psi_{k}$ as the *k*-th element of the orthonormal basis

(4) $$\Psi =\{\psi_{k}\in L_{2}\left(\Omega \right)\;|\;k\in N_{0},\;∥\psi_{k}{∥}_{v}=1\wedge {\langle \psi_{k},\psi_{j}\rangle }_{v}=0\;\mathrm{for}\;k\neq j\}$$

with respect to the function space $X\subseteq L_{2}\left(\Omega \right)$ with the positive weight function v:Ω→R:

(5) $$\begin{array}{c} \begin{array}{cc} X: & =\{f:\Omega \to R,∥f{∥}_{v}^{2}<\infty \}=\mathrm{span}\left(\Psi \right)\;,\\ {\langle f,g\rangle }_{v} & =\int_{\Omega }v\left(x\right)f\left(x\right)g\left(x\right)dx\;. \end{array} \end{array}$$

The weight *v* serves as a degree of freedom within the upcoming problem-solving process. A reduced basis is utilized for approximating the solution space by exploiting orthonormality with respect to the spatial component resulting in a boundary estimation by algebraic means.

### 2.3. Modulating Functions

An extension of the modulating functions method to distributed systems can be obtained by defining the kernel function in both time and spatial domain [22,24].

**Definition** **1** (Modulation Functional)**.** *The state modulation functional is defined by*

(6) $$M\left[h\right]=\int_{t-T}^{t}\int_{0}^{L}\varphi \left(x,\tau -t+T\right)h\left(x,\tau \right)dxd\tau$$

*where h: $\left[0,L\right]\times R_{0}^{+}\to R$ and φ: [0,L]×[0,T]→R is the modulating function to be constructed.*

For simplicity the inner product notation

$$\begin{array}{c} {\langle \varphi ,h\rangle }_{\Omega ,I}:=M\left[h\right] \end{array}$$

is used where the moving time horizon is denoted by I:=[t−T,t] with the receding horizon length T>0, also called the time window. If the integration only concerns the temporal or spatial variable, ${\langle \varphi ,h\rangle }_{I}$ and ${\langle \varphi ,h\rangle }_{\Omega }$ are used. In the following, an application of the operator (6) leads to a similar construction of conditions for the modulation kernel φ with a more systematic auxiliary problem than in the ODE case.

## 3. Non-Asymptotic Observer Design for Coupled PDE System

The major research goal of the contribution is related to the extension of the non-asymptotic observer design approach for PDEs by using modulating functions from [23] towards coupled PDE systems. In order to construct a relationship between the known measurements and the parameterization of the unmeasured state variable, the modulation functional is applied to the dynamics of the system and suitable conditions for neglecting unknown terms are formulated. To this end, a system of auxiliary equations is built for calculating the modulation kernels offline which resemble the form of the original system in an adjoint way. The whole operation results in an estimator equation for obtaining the series expansion coefficients that solely relies on an integration-based time filter operation on the sensor data.

### 3.1. Derivation of the Auxiliary System

For the derivation of the auxiliary system, the modulation functional (6) is applied to the dynamics of the system (1). The following calculations are performed in a similar fashion as in [23]. First, the modulation functional is applied to the second spatial derivative of the state with φ as the modulation function defined in (6). Considering each particular derivative for every state component $w_{l}\left(x,t\right)$ of *W* with l∈{1,…,n} and using derivation by parts leads to

$$\begin{array}{c} {\langle \varphi ,\frac{\partial^{2}w_{l}}{\partial x^{2}}\rangle }_{\Omega ,I}=\int_{t-T}^{t}\varphi \left(x,\tau -t+T\right)\frac{\partial w_{l}}{\partial x}\left(x,\tau \right)d\tau {|}_{x=0}^{x=L}-\int_{t-T}^{t}\frac{\partial \varphi }{\partial x}\left(x,\tau -t+T\right)w_{l}\left(x,\tau \right)d\tau {|}_{x=0}^{x=L}+{\langle \frac{\partial^{2}\varphi }{\partial x^{2}},w_{l}\rangle }_{\Omega ,I} \end{array}$$

(7) $$\begin{array}{c} \Rightarrow \;{\langle \varphi ,\frac{\partial^{2}w_{l}}{\partial x^{2}}\rangle }_{\Omega ,I}=\int_{t-T}^{t}\left(M_{L}^{2}+M_{0}^{2}\right)\left[\varphi ,w_{l}\right]d\tau +{\langle \frac{\partial^{2}\varphi }{\partial x^{2}},w_{l}\rangle }_{\Omega ,I} \end{array}$$

with the substituted function kernel operations

$$\begin{array}{cc} M_{L}^{2}\left[\varphi ,w_{l}\right] & =\varphi \left(L,\tau -t+T\right)\frac{\partial w_{l}}{\partial x}\left(L,\tau \right)-\frac{\partial \varphi }{\partial x}\left(L,\tau -t+T\right)w_{l}\left(L,\tau \right)\\ M_{0}^{2}\left[\varphi ,w_{l}\right] & =-\varphi \left(0,\tau -t+T\right)\frac{\partial w_{l}}{\partial x}\left(0,\tau \right)+\frac{\partial \varphi }{\partial x}\left(0,\tau -t+T\right)w_{l}\left(0,\tau \right). \end{array}$$

Now, the modulation functional is applied to the first spatial derivative of the state. Using derivation by parts again we get

(8) $$\begin{array}{cc} {\langle \varphi ,\frac{\partial w_{l}}{\partial x}\rangle }_{\Omega ,I} & =\int_{t-T}^{t}\varphi \left(x,\tau -t+T\right)w_{l}\left(x,\tau \right){|}_{x=0}^{x=L}+{\langle \frac{\partial \varphi }{\partial x},w_{l}\rangle }_{\Omega ,I}=\int_{t-T}^{t}\left(M_{L}^{1}+M_{0}^{1}\right)\left(\varphi ,w_{l}\right)d\tau +{\langle \frac{\partial \varphi }{\partial x},w_{l}\rangle }_{\Omega ,I} \end{array}$$

where

$$\begin{array}{cc} M_{L}^{1}\left(\varphi ,w_{l}\right) & =\varphi \left(L,\tau -t+T\right)w_{l}\left(L,\tau \right)\\ M_{0}^{1}\left(\varphi ,w_{l}\right) & =-\varphi \left(0,\tau -t+T\right)w_{l}\left(0,\tau \right). \end{array}$$

Furthermore, the modulation functional is applied to the time derivative of the state. Similarly, using derivation by parts leads to

$$\begin{array}{cc} {\langle \varphi ,\frac{\partial w_{l}}{\partial t}\rangle }_{\Omega ,I} & =\int_{0}^{L}\varphi \left(x,\tau -t+T\right)w_{l}\left(x,\tau \right)dx{|}_{\tau =t-T}^{\tau =t}-{\langle \frac{\partial \varphi }{\partial \tau },w_{l}\rangle }_{\Omega ,I}. \end{array}$$

Selecting the following initial and final temporal conditions for the modulating function with reduced function basis $\left\{\psi_{m},m\in 0,1,\ldots ,N\right\}$, N∈N, from (4) for estimation, i.e.,

(9) $$\left\{\begin{array}{cc} \varphi^{m}\left(x,0\right) & =0\\ \varphi^{m}\left(x,T\right) & =v\left(x\right)\psi_{m}\left(x\right) \end{array}\right.$$

we use the basis expansion from (3) and the spatial integration term becomes

$$\begin{array}{c} \int_{0}^{L}\varphi^{m}\left(x,\tau -t+T\right)w_{l}\left(x,\tau \right)dx{|}_{\tau =t-T}^{\tau =t}=\int_{0}^{L}\varphi^{m}\left(x,T\right)w_{l}\left(x,t\right)dx=\sum_{i=0}^{\infty }c_{l}^{i}\left(t\right){\langle \psi_{m},\psi_{i}\rangle }_{v}=c_{l}^{m}\left(t\right). \end{array}$$

Finally, the conditions of (9) establish the relation

(10) $$\begin{array}{cc} {\langle \varphi^{m},\frac{\partial w_{l}}{\partial t}\rangle }_{\Omega ,I} & =c_{l}^{m}\left(t\right)-{\langle \frac{\partial \varphi^{m}}{\partial \tau },w_{l}\rangle }_{\Omega ,I}. \end{array}$$

Applying the modulating functions $\varphi_{i}^{m},i\in \left\{1,\ldots ,n\right\}$, to the *i*-th equation of (1) and utilizing the former calculations we obtain

$$\begin{array}{c} {\langle \varphi_{i}^{m},\frac{\partial w_{i}}{\partial t}\rangle }_{\Omega ,I}=\sum_{j=1}^{n}\langle \varphi_{i}^{m},\epsilon_{ij}\left(x\right)\frac{\partial^{2}w_{j}}{\partial x^{2}}\rangle +\sum_{j=1}^{n}\langle \varphi_{i}^{m},\phi_{ij}\left(x\right)\frac{\partial w_{j}}{\partial x}\rangle +\sum_{j=1}^{n}\langle \varphi_{i}^{m},\lambda_{ij}\left(x\right)w_{j}\rangle \end{array}$$

which using the associative properties of the modulation functions yields

$$\begin{array}{c} {\langle \varphi_{i}^{m},\frac{\partial w_{i}}{\partial t}\rangle }_{\Omega ,I}=\sum_{j=1}^{n}\langle \epsilon_{ij}\left(x\right)\varphi_{i}^{m},\frac{\partial^{2}w_{j}}{\partial x^{2}}\rangle +\sum_{j=1}^{n}\langle \phi_{ij}\left(x\right)\varphi_{i}^{m},\frac{\partial w_{j}}{\partial x}\rangle +\sum_{j=1}^{n}\langle \lambda_{ij}\left(x\right)\varphi_{i}^{m},w_{j}\rangle . \end{array}$$

From the previous results, (7), (8) and (10), we first draw

$$\begin{array}{c} \begin{array}{c} c_{i}^{m}\left(t\right)={\langle \frac{\partial \varphi_{i}^{m}}{\partial \tau },w_{i}\rangle }_{\Omega ,I}+\sum_{j=1}^{n}\int_{t-T}^{t}\left(M_{L}^{2}+M_{0}^{2}\right)\left[\epsilon_{ij}\left(x\right)\varphi_{i}^{m},w_{j}\right]+\left(M_{L}^{1}+M_{0}^{1}\right)\left[\phi_{ij}\left(x\right)\varphi_{i}^{m},w_{j}\right]d\tau \\ +\sum_{j=1}^{n}{\langle \frac{\partial^{2}\left(\epsilon_{ij}\left(x\right)\varphi_{i}^{m}\right)}{\partial x^{2}}-\frac{\partial (\phi_{ij}\left(x\right)\varphi_{i}^{m})}{\partial x}+\lambda_{ij}\left(x\right)\varphi_{i}^{m},w_{j}\rangle }_{\Omega ,I} \end{array} \end{array}$$

that by expanding the spatial derivations in the left part leads to

(11) $$\begin{array}{c} \begin{array}{c} c_{i}^{m}\left(t\right)={\langle \frac{\partial \varphi_{i}^{m}}{\partial \tau },w_{i}\rangle }_{\Omega ,I}+\sum_{j=1}^{n}\int_{t-T}^{t}\left(M_{L}^{2}+M_{0}^{2}\right)\left[\epsilon_{ij}\left(x\right)\varphi_{i}^{m},w_{j}\right]+\left(M_{L}^{1}+M_{0}^{1}\right)\left[\phi_{ij}\left(x\right)\varphi_{i}^{m},w_{j}\right]d\tau \\ +\sum_{j=1}^{n}{\langle \epsilon_{ij}\left(x\right)\frac{\partial^{2}\varphi_{i}^{m}}{\partial x^{2}}+\left(2\frac{\partial \epsilon_{ij}}{\partial x}-\phi_{ij}\right)\left(x\right)\frac{\partial \varphi_{i}^{m}}{\partial x}+\left(\frac{\partial^{2}\epsilon_{ij}}{\partial x^{2}}-\frac{\partial \phi_{ij}}{\partial x}+\lambda_{ij}\right)\left(x\right)\varphi_{i}^{m},w_{j}\rangle }_{\Omega ,I}. \end{array} \end{array}$$

Up to this part, the procedure is applied along [23]. It is worth noticing that for eliminating the part of the inner product brackets multiplied with the unknown state $w_{j}$, *n* auxiliary PDE equations would have to be solved for $\varphi_{i}^{m}$ with only one solution. As this is an ill-posed problem, more equations need to be generated. Multiplying every line of (11) by $k_{i}\in R$ and summing up the components first generates the set of equations

$$\begin{array}{c} \sum_{i=1}^{n}k_{i}c_{i}^{m}\left(t\right)=\sum_{i,j=1}^{n}k_{i}\int_{t-T}^{t}\left(M_{L}^{2}+M_{0}^{2}\right)\left[\epsilon_{ij}\left(x\right)\varphi_{i}^{m},w_{j}\right]+\left(M_{L}^{1}+M_{0}^{1}\right)\left[\phi_{ij}\left(x\right)\varphi_{i}^{m},w_{j}\right]d\tau \\ +\sum_{i=1}^{n}k_{i}{\langle \frac{\partial \varphi_{i}^{m}}{\partial \tau },w_{i}\rangle }_{\Omega ,I}+\sum_{i=1}^{n}k_{i}\sum_{j=1}^{n}{\langle \epsilon_{ij}\left(x\right)\frac{\partial^{2}\varphi_{i}^{m}}{\partial x^{2}}+\left(2\frac{\partial \epsilon_{ij}}{\partial x}-\phi_{ij}\right)\left(x\right)\frac{\partial \varphi_{i}^{m}}{\partial x}+\left(\frac{\partial^{2}\epsilon_{ij}}{\partial x^{2}}-\frac{\partial \phi_{ij}}{\partial x}+\lambda_{ij}\right)\left(x\right)\varphi_{i}^{m},w_{j}\rangle }_{\Omega ,I} \end{array}$$

which taking $k_{i}$ into the brackets and switching the index *i* with *j* in the last sum gives

$$\begin{array}{c} \sum_{i=1}^{n}k_{i}c_{i}^{m}\left(t\right)=\sum_{i,j=1}^{n}k_{i}\int_{t-T}^{t}\left(M_{L}^{2}+M_{0}^{2}\right)\left[\epsilon_{ij}\left(x\right)\varphi_{i}^{m},w_{j}\right]+\left(M_{L}^{1}+M_{0}^{1}\right)\left[\phi_{ij}\left(x\right)\varphi_{i}^{m},w_{j}\right]d\tau \\ +\sum_{i=1}^{n}k_{i}{\langle \frac{\partial \varphi_{i}^{m}}{\partial \tau },w_{i}\rangle }_{\Omega ,I}+\sum_{i,j=1}^{n}{\langle k_{j}\epsilon_{ji}\left(x\right)\frac{\partial^{2}\varphi_{i}^{m}}{\partial x^{2}}+k_{j}\left(2\frac{\partial \epsilon_{ji}}{\partial x}-\phi_{ji}\right)\left(x\right)\frac{\partial \varphi_{i}^{m}}{\partial x}+k_{j}\left(\frac{\partial^{2}\epsilon_{ji}}{\partial x^{2}}-\frac{\partial \phi_{ji}}{\partial x}+\lambda_{ji}\right)\left(x\right)\varphi_{i}^{m},w_{i}\rangle }_{\Omega ,I}. \end{array}$$

Then, first exchanging the order of summation leads to

$$\begin{array}{c} \sum_{i=1}^{n}k_{i}c_{i}^{m}\left(t\right)=\sum_{i,j=1}^{n}k_{i}\int_{t-T}^{t}\left(M_{L}^{2}+M_{0}^{2}\right)\left[\epsilon_{ij}\left(x\right)\varphi_{i}^{m},w_{j}\right]+\left(M_{L}^{1}+M_{0}^{1}\right)\left[\phi_{ij}\left(x\right)\varphi_{i}^{m},w_{j}\right]d\tau \\ +\sum_{i=1}^{n}{\langle k_{i}\frac{\partial \varphi_{i}^{m}}{\partial \tau }+\sum_{j=1}^{n}\left(k_{j}\epsilon_{ji}\left(x\right)\frac{\partial^{2}\varphi_{i}^{m}}{\partial x^{2}}+k_{j}\left(2\frac{\partial \epsilon_{ji}}{\partial x}-\phi_{ji}\right)\left(x\right)\frac{\partial \varphi_{i}^{m}}{\partial x}+k_{j}\left(\frac{\partial^{2}\epsilon_{ji}}{\partial x^{2}}-\frac{\partial \phi_{ji}}{\partial x}+\lambda_{ji}\right)\left(x\right)\varphi_{i}^{m}\right),w_{i}\rangle }_{\Omega ,I}, \end{array}$$

finally, dividing and multiplying the term within the inner product brackets by $k_{i}\neq 0$ to

(12) $$\begin{array}{c} \sum_{i=1}^{n}k_{i}c_{i}^{m}\left(t\right)=\sum_{i,j=1}^{n}k_{i}\int_{t-T}^{t}\left(M_{L}^{2}+M_{0}^{2}\right)\left[\epsilon_{ij}\left(x\right)\varphi_{i}^{m},w_{j}\right]+\left(M_{L}^{1}+M_{0}^{1}\right)\left[\phi_{ij}\left(x\right)\varphi_{i}^{m},w_{j}\right]d\tau \\ +\sum_{i=1}^{n}k_{i}{\langle \frac{\partial \varphi_{i}^{m}}{\partial \tau }+\sum_{j=1}^{n}\left(\frac{k_{j}}{k_{i}}\epsilon_{ji}\left(x\right)\frac{\partial^{2}\varphi_{i}^{m}}{\partial x^{2}}+\frac{k_{j}}{k_{i}}\left(2\frac{\partial \epsilon_{ji}}{\partial x}-\phi_{ji}\right)\left(x\right)\frac{\partial \varphi_{i}^{m}}{\partial x}+\frac{k_{j}}{k_{i}}\left(\frac{\partial^{2}\epsilon_{ji}}{\partial x^{2}}-\frac{\partial \phi_{ji}}{\partial x}+\lambda_{ji}\right)\left(x\right)\varphi_{i}^{m}\right),w_{i}\rangle }_{\Omega ,I}. \end{array}$$

For now, it is assumed that the terms $M_{L}^{2},M_{0}^{2},M_{L}^{1},M_{0}^{1}$ can be calculated (are known). This is demonstrated in Section 3.2. In order to annihilate the term in brackets and thus, leaving the left part of (12) with only known terms, the following condition is needed:

$$\begin{array}{c} -\frac{\partial \varphi_{i}^{m}}{\partial \tau }=\sum_{j=1}^{n}\frac{k_{j}}{k_{i}}\epsilon_{ji}\left(x\right)\frac{\partial^{2}\varphi_{i}^{m}}{\partial x^{2}}+\sum_{j=1}^{n}\frac{k_{j}}{k_{i}}\left(2\frac{\partial \epsilon_{ji}}{\partial x}-\phi_{ji}\right)\left(x\right)\frac{\partial \varphi_{i}^{m}}{\partial x}+\sum_{j=1}^{n}\frac{k_{j}}{k_{i}}\left(\frac{\partial^{2}\epsilon_{ji}}{\partial x^{2}}-\frac{\partial \phi_{ji}}{\partial x}+\lambda_{ji}\right)\left(x\right)\varphi_{i}^{m},i=1,\cdots ,n. \end{array}$$

The following adjoint coupled PDE is constructed to determine the modulating functions:

$$\left\{\begin{array}{cc} -\frac{\partial \varphi^{m}}{\partial \tau }\left(x,\tau \right) & =\bar{\Sigma }\left(x\right)\frac{\partial^{2}\varphi^{m}}{\partial x^{2}}\left(x,\tau \right)+\bar{\Phi }\left(x\right)\frac{\partial \varphi^{m}}{\partial x}\left(x,\tau \right)+\bar{\Lambda }\left(x\right)\varphi^{m}\left(x,\tau \right)\\ \bar{\Sigma }\left(x\right) & =[\frac{k_{j}}{k_{i}}\epsilon_{ji}\left(x\right)]\\ \bar{\Phi }\left(x\right) & =[\frac{k_{j}}{k_{i}}\left(2\frac{\partial \epsilon_{ji}}{\partial x}-\phi_{ji}\right)\left(x\right)]\\ \bar{\Lambda }\left(x\right) & =[\frac{k_{j}}{k_{i}}\left(\frac{\partial^{2}\epsilon_{ji}}{\partial x^{2}}-\frac{\partial \phi_{ji}}{\partial x}+\lambda_{ji}\right)\left(x\right)] \end{array}\right.$$

with τ∈[0,T] and the initial and final condition given by (9).

The boundary conditions remain degrees of freedom. However, the main challenge is that the final condition needs to be met precisely. To this end, the following boundary condition is added to the auxiliary problem as an extra degree of freedom:

$$\varphi^{m}\left(0,\tau \right)=\eta^{m}\left(\tau \right).$$

Note that the negative sign in front of the spatial derivative implies non-causal nature of the distributed dynamics. For that reason, a transformation to forward time is used similar to [23] with σ∈[0,T]:

(13) $$\xi^{m}\left(x,\sigma \right):=\varphi^{m}\left(x,T-\sigma \right).$$

This results in a transformed auxiliary problem with an added boundary condition for the signal model control to fulfill the specifications

(14) $$\left\{\begin{array}{cc} \frac{\partial \xi^{m}}{\partial \sigma }\left(x,\sigma \right) & =\bar{\Sigma }\left(x\right)\frac{\partial^{2}\xi^{m}}{\partial x^{2}}\left(x,\sigma \right)+\bar{\Phi }\left(x\right)\frac{\partial \xi^{m}}{\partial x}\left(x,\sigma \right)+\bar{\Lambda }\left(x\right)\xi^{m}\left(x,\sigma \right)\\ \xi^{m}\left(x,0\right) & =v\left(x\right)\psi^{m}\left(x\right)\\ \xi^{m}\left(x,T\right) & =0\\ \xi^{m}\left(0,\sigma \right) & ={\tilde{\eta }}^{m}\left(\sigma \right). \end{array}\right.$$

The specified goal of $\xi^{m}\left(x,T\right)=0$ is the main reason for the added boundary condition for the auxiliary model control ${\tilde{\eta }}^{m}$. This specification implies that the auxiliary model has to be stabilized to zero within the time window of length *T*. If that is not achieved, the approximation of the coefficient $c_{j}^{m}$ includes a significant error and in consequence the estimation has an induced error. As a consequence, (14) implies that (12) becomes

(15) $$\begin{array}{c} \sum_{i=1}^{n}k_{i}c_{i}^{m}\left(t\right)=\sum_{i,j=1}^{n}k_{i}\int_{t-T}^{t}\left(M_{L}^{2}+M_{0}^{2}\right)\left[\epsilon_{ij}\left(x\right)\varphi_{i}^{m},w_{j}\right]+\left(M_{L}^{1}+M_{0}^{1}\right)\left[\phi_{ij}\left(x\right)\varphi_{i}^{m},w_{j}\right]d\tau . \end{array}$$

In order to determine each $c_{i}^{m}$ value, further (n−1) equations can be generated similar to (15) with different $k_{i}$ to form a system of *n* equations. Since (12) holds true for any of these relations, we get

(16) $$\begin{array}{c} \sum_{i=1}^{n}k_{hi}c_{i}^{m}\left(t\right)=\sum_{i,j=1}^{n}k_{hi}\int_{t-T}^{t}\left(M_{L}^{2}+M_{0}^{2}\right)\left[\epsilon_{ij}\left(x\right)\varphi_{i}^{m},w_{j}\right]+\left(M_{L}^{1}+M_{0}^{1}\right)\left[\phi_{ij}\left(x\right)\varphi_{i}^{m},w_{j}\right]d\tau ,\;h=1,\cdots ,n. \end{array}$$

If the function basis approximation order is *N*, then N+1 systems exist with the form of (14). In total, n(N+1) auxiliary systems are generated and need to be solved, and $n{\left(N+1\right)}^{2}$ modulating functions result in total. It is also worth noticing that after solving (14), an inverse time transformation has to be made to obtain the modulating functions $\varphi_{i}^{m}$. This transformation is

(17) $$\varphi^{m}\left(x,\sigma \right):=\xi^{m}\left(x,T-\sigma \right).$$

For the reconstruction of the states, it has to be ensured that the auxiliary system requirements are fulfilled. Once the auxiliary problem is solved, after the inverse time transformation (17), the modulating functions $\varphi_{hi}^{m}$ for 1≤h,i≤n and 0≤m≤N are obtained. Then (16) can be rewritten in a linear system of equations as per

$$\begin{array}{c} \left[\begin{array}{ccc} k_{h1} & \ldots & k_{hn} \end{array}\right]\left[\begin{array}{c} c_{1}^{m}\left(t\right)\\ \vdots \\ c_{n}^{m}\left(t\right) \end{array}\right]=M_{h}^{m}\left(t\right) \end{array}$$

where

(18) $$\begin{array}{c} M_{h}^{m}\left(t\right)=\sum_{i=1}^{n}\sum_{j=1}^{n}k_{hi}\int_{t-T}^{t}\left(M_{L}^{2}+M_{0}^{2}\right)\left[\epsilon_{ij}\left(x\right)\varphi_{hi}^{m},w_{j}\right]+\left(M_{L}^{1}+M_{0}^{1}\right)\left[\phi_{ij}\left(x\right)\varphi_{hi}^{m},w_{j}\right]d\tau \;. \end{array}$$

Using

$$\begin{array}{c} K=\left[\begin{array}{ccc} k_{11} & \ldots & k_{1n}\\ \vdots & \ddots & \vdots \\ k_{n1} & \ldots & k_{nn} \end{array}\right],C\left(t\right)=\left[\begin{array}{ccc} c_{1}^{0}\left(t\right) & \ldots & c_{1}^{N}\left(t\right)\\ \vdots & \ddots & \vdots \\ c_{n}^{0}\left(t\right) & \ldots & c_{n}^{N}\left(t\right) \end{array}\right]\;, \end{array}$$

a decoupling follows if *K* is invertible, thus

(19) $$\begin{array}{c} C\left(t\right)=K^{-1}\left[\begin{array}{ccc} M_{1}^{0}\left(t\right) & \ldots & M_{1}^{N}\left(t\right)\\ \vdots & \ddots & \vdots \\ M_{n}^{0}\left(t\right) & \ldots & M_{n}^{N}\left(t\right) \end{array}\right]=K^{-1}M\left(t\right). \end{array}$$

With these coefficients, the state can be reconstructed using the function expansion representation from (3):

(20) $$\begin{array}{cc} W(x,t) & =\left[\begin{array}{c} \sum_{k=0}^{\infty }c_{1}^{k}\left(t\right)\psi_{k}\left(x\right)\\ \vdots \\ \sum_{k=0}^{\infty }c_{n}^{k}\left(t\right)\psi_{k}\left(x\right) \end{array}\right]\approx \left[\begin{array}{c} \sum_{k=0}^{N}c_{1}^{k}\left(t\right)\psi_{k}\left(x\right)\\ \vdots \\ \sum_{k=0}^{N}c_{n}^{k}\left(t\right)\psi_{k}\left(x\right) \end{array}\right]=C\left(t\right)\Psi \left(x\right)=K^{-1}M\left(t\right)\Psi \left(x\right). \end{array}$$

With this expression the state can be reconstructed, solving the estimation problem. It shall be noted that for the reconstruction of state, the calculation of the coefficients of the basis expansion is required. The calculation follows (19) and depends on matrix *K* and M.

The main idea for the procedure is to use a different modulating function for every equation of the system. Then, to overcome the coupling that exists in the system, all the equations are added up to get the coupling into the modulating function equation. Finally, more coupled systems can be constructed by adding up the equations but with different constants.

### 3.2. Calculation of the Modulation Operators

Now, $M_{L}^{2},M_{L}^{1},M_{0}^{2}$, and $M_{0}^{1}$ are calculated from known terms. Without loss of generality, it is assumed that $x^{*}=0$. Then, the following system of equations is obtained:

$$\begin{array}{c} P_{1}\frac{\partial W}{\partial x}\left(0,t\right)+P_{0}W\left(0,t\right)=F\left(t\right)\\ R_{1}\frac{\partial W}{\partial x}\left(0,t\right)+R_{0}W\left(0,t\right)=Y\left(t\right). \end{array}$$

If $P_{1}$ and $R_{1}$ or $P_{0}$ and $R_{0}$ are linearly independent, then W(0,t) and $\frac{\partial W}{\partial x}\left(0,t\right)$ satisfy

(21) $$\left[\begin{array}{c} \frac{\partial W}{\partial x}\left(0,t\right)\\ W(0,t) \end{array}\right]={\left[\begin{array}{cc} P_{1} & P_{0}\\ R_{1} & R_{0} \end{array}\right]}^{-1}\left[\begin{array}{c} F(t)\\ Y(t) \end{array}\right].$$

With additional knowledge of the modulating function φ, $M_{0}^{2}$ and $M_{0}^{1}$ are known and can be calculated from (18), leading to

$$\begin{array}{c} \sum_{i,j=1}^{n}k_{hi}\int_{t-T}^{t}\left(M_{0}^{2}\left[\epsilon_{ij}\left(x\right)\varphi_{hi}^{m},w_{j}\right]+M_{0}^{1}\left[\phi_{ij}\left(x\right)\varphi_{hi}^{m},w_{j}\right]\right)d\tau =\sum_{i,j=1}^{n}k_{hi}\int_{t-T}^{t}(-\left(\epsilon_{ij}\varphi_{hi}^{m}\right)\left(0,\tau -t+T\right)\frac{\partial w_{j}}{\partial x}\left(0,\tau \right)\\ +\left(\frac{\partial (\epsilon_{ij}\varphi_{hi}^{m})}{\partial x}-\phi_{ij}\varphi_{hi}^{m}\right)\left(0,\tau -t+T\right)w_{j}\left(0,\tau \right))d\tau . \end{array}$$

Arranging the equation in a vectorial form

(22) $$\begin{array}{c} \sum_{i,j=1}^{n}k_{hi}\int_{t-T}^{t}\left(M_{0}^{2}\left[\epsilon_{ij}\left(x\right)\varphi_{hi}^{m},w_{j}\right]+M_{0}^{1}\left[\phi_{ij}\left(x\right)\varphi_{hi}^{m},w_{j}\right]\right)d\tau =\\ \int_{t-T}^{t}\left[\begin{array}{cc} -{\tilde{\Sigma }}_{h}\left(0,\tau -t+T\right) & \left(\frac{\partial {\tilde{\Sigma }}_{h}}{\partial x}-{\tilde{\Phi }}_{h}\right)\left(0,\tau -t+T\right) \end{array}\right]\left[\begin{array}{c} \frac{\partial W}{\partial x}\left(0,\tau \right)\\ W(0,\tau ) \end{array}\right]d\tau \end{array}$$

where

$$\begin{array}{c} {\tilde{\Sigma }}_{h}\left(x,\tau -t+T\right)={\left\{k_{hi}\left(\varphi_{hi}\epsilon_{ij}\right)\left(x,\tau -t+T\right)\right\}}_{1\leq i,j\leq n}\\ {\tilde{\Phi }}_{h}\left(x,\tau -t+T\right)={\left\{k_{hi}\left(\phi_{ij}\varphi_{hi}\right)\left(x,\tau -t+T\right)\right\}}_{1\leq i,j\leq n}. \end{array}$$

and using (21) yields

(23) $$\begin{array}{c} \sum_{i,j=1}^{n}k_{hi}\int_{t-T}^{t}\left(M_{0}^{2}\left[\epsilon_{ij}\left(x\right)\varphi_{hi}^{m},w_{j}\right]+M_{0}^{1}\left[\phi_{ij}\left(x\right)\varphi_{hi}^{m},w_{j}\right]\right)d\tau =\\ \int_{t-T}^{t}\left[\begin{array}{cc} -{\tilde{\Sigma }}_{h}\left(0,\tau -t+T\right) & \left(\frac{\partial {\tilde{\Sigma }}_{h}}{\partial x}-{\tilde{\Phi }}_{h}\right)\left(0,\tau -t+T\right) \end{array}\right]{\left[\begin{array}{cc} P_{1} & P_{0}\\ R_{1} & R_{0} \end{array}\right]}^{-1}\left[\begin{array}{c} F(\tau )\\ Y(\tau ) \end{array}\right]d\tau . \end{array}$$

For $M_{L}^{2},M_{L}^{1}$, the equation can be rewritten similarly:

(24) $$\begin{array}{c} \sum_{i,j=1}^{n}k_{hi}\int_{t-T}^{t}\left(M_{L}^{2}\left[\epsilon_{ij}\left(x\right)\varphi_{hi}^{m},w_{j}\right]+M_{L}^{1}\left[\phi_{ij}\left(x\right)\varphi_{hi}^{m},w_{j}\right]\right)d\tau =\\ \int_{t-T}^{t}\left({\tilde{\Sigma }}_{h}\left(L,\tau -t+T\right)W_{x}\left(L,\tau \right)-\left(\frac{\partial {\tilde{\Sigma }}_{h}}{\partial x}+{\tilde{\Phi }}_{h}\right)\left(L,\tau -t+T\right)W\left(L,\tau \right)\right)d\tau . \end{array}$$

From here, (2) can be used only involving known terms.

If $Q_{1}$ is invertible, then $\frac{\partial W}{\partial x}\left(L,t\right)=Q_{1}^{-1}\left(U\left(t\right)-Q_{0}W\left(L,t\right)\right)$ and using (24) results in

$$\begin{array}{c} \sum_{i,j=1}^{n}k_{hi}\int_{t-T}^{t}\left(M_{L}^{2}\left[\epsilon_{ij}\left(x\right)\varphi_{hi}^{m},w_{j}\right]+M_{L}^{1}\left[\phi_{ij}\left(x\right)\varphi_{hi}^{m},w_{j}\right]\right)d\tau \\ =\int_{t-T}^{t}\left({\tilde{\Sigma }}_{h}\left(L,\tau -t+T\right)Q_{1}^{-1}U\left(\tau \right)-\left(\left(\frac{\partial {\tilde{\Sigma }}_{h}}{\partial x}+{\tilde{\Phi }}_{h}\right)\left(L,\tau -t+T\right)+{\tilde{\Sigma }}_{h}\left(L,\tau -t+T\right)Q_{1}^{-1}Q_{0}\right)W\left(L,\tau \right)\right)d\tau . \end{array}$$

and imposing

(25) $$\left(\frac{\partial {\tilde{\Sigma }}_{h}}{\partial x}+{\tilde{\Phi }}_{h}\right)\left(L,\tau -t+T\right)+{\tilde{\Sigma }}_{h}\left(L,\tau -t+T\right)Q_{1}^{-1}Q_{0}=0$$

then

$$\begin{array}{c} \sum_{i,j=1}^{n}k_{hi}\int_{t-T}^{t}\left(M_{L}^{2}\left[\epsilon_{ij}\left(x\right)\varphi_{hi}^{m},w_{j}\right]+M_{L}^{1}\left[\phi_{ij}\left(x\right)\varphi_{hi}^{m},w_{j}\right]\right)d\tau =\int_{t-T}^{t}\left({\tilde{\Sigma }}_{h}\left(L,\tau -t+T\right)Q_{1}^{-1}U\left(\tau \right)\right)d\tau \;. \end{array}$$

Finally, in addition to (23) we have

$$\begin{array}{c} \sum_{i,j=1}^{n}k_{hi}\int_{t-T}^{t}\left(M_{L}^{2}+M_{L}^{1}\right)\left[\epsilon_{ij}\left(x\right)\varphi_{hi}^{m},w_{j}\right]+\left(M_{0}^{2}+M_{0}^{1}\right)\left[\phi_{ij}\left(x\right)\varphi_{hi}^{m},w_{j}\right]d\tau \\ =\int_{t-T}^{t}\left[\begin{array}{cc} -{\tilde{\Sigma }}_{h}\left(0,\tau -t+T\right) & \left(\frac{\partial {\tilde{\Sigma }}_{h}}{\partial x}-{\tilde{\Phi }}_{h}\right)\left(0,\tau -t+T\right) \end{array}\right]{\left[\begin{array}{cc} P_{1} & P_{0}\\ R_{1} & R_{0} \end{array}\right]}^{-1}\left[\begin{array}{c} F(\tau )\\ Y(\tau ) \end{array}\right]d\tau \\ +\int_{t-T}^{t}\left({\tilde{\Sigma }}_{h}\left(L,\tau -t+T\right)Q_{1}^{-1}U\left(\tau \right)\right)d\tau \;. \end{array}$$

If $Q_{0}$ is invertible, then $W\left(L,t\right)=Q_{0}^{-1}\left(U\left(t\right)-Q_{1}\frac{\partial W}{\partial x}\left(L,t\right)\right)$ and hence

$$\begin{array}{c} \sum_{i,j=1}^{n}k_{hi}\int_{t-T}^{t}M_{L}^{2}\left[\epsilon_{ij}\left(x\right)\varphi_{hi}^{m},w_{j}\right]+M_{L}^{1}\left[\phi_{ij}\left(x\right)\varphi_{hi}^{m},w_{j}\right]d\tau \\ =\int_{t-T}^{t}-\left(\left(\frac{\partial {\tilde{\Sigma }}_{h}}{\partial x}+{\tilde{\Phi }}_{h}\right)\left(L,\tau -t+T\right)Q_{0}^{-1}U\left(\tau \right)\right)+\left({\tilde{\Sigma }}_{h}\left(L,\tau -t+T\right)+\left(\frac{\partial {\tilde{\Sigma }}_{h}}{\partial x}+{\tilde{\Phi }}_{h}\right)\left(L,\tau -t+T\right)Q_{0}^{-1}Q_{1}\right)W_{x}\left(L,\tau \right)d\tau \end{array}$$

and imposing

(26) $${\tilde{\Sigma }}_{h}\left(L,\tau -t+T\right)+\left(\frac{\partial {\tilde{\Sigma }}_{h}}{\partial x}+{\tilde{\Phi }}_{h}\right)\left(L,\tau -t+T\right)Q_{0}^{-1}Q_{1}=0$$

then

$$\begin{array}{c} \sum_{i,j=1}^{n}k_{hi}\int_{t-T}^{t}\left(M_{L}^{2}\left[\epsilon_{ij}\left(x\right)\varphi_{hi}^{m},w_{j}\right]+M_{L}^{1}\left[\phi_{ij}\left(x\right)\varphi_{hi}^{m},w_{j}\right]\right)d\tau =-\int_{t-T}^{t}\left(\left(\frac{\partial {\tilde{\Sigma }}_{h}}{\partial x}+{\tilde{\Phi }}_{h}\right)\left(L,\tau -t+T\right)Q_{0}^{-1}U\left(\tau \right)\right)d\tau \;. \end{array}$$

Finally, in addition to (23) we have

$$\begin{array}{c} \sum_{i,j=1}^{n}k_{hi}\int_{t-T}^{t}\left(M_{L}^{2}+M_{L}^{1}\right)\left[\epsilon_{ij}\left(x\right)\varphi_{hi}^{m},w_{j}\right]+\left(M_{0}^{2}+M_{0}^{1}\right)\left[\phi_{ij}\left(x\right)\varphi_{hi}^{m},w_{j}\right])d\tau \\ =\int_{t-T}^{t}\left[\begin{array}{cc} -{\tilde{\Sigma }}_{h}\left(0,\tau -t+T\right) & \left(\frac{\partial {\tilde{\Sigma }}_{h}}{\partial x}-{\tilde{\Phi }}_{h}\right)\left(0,\tau -t+T\right) \end{array}\right]{\left[\begin{array}{cc} P_{1} & P_{0}\\ R_{1} & R_{0} \end{array}\right]}^{-1}\left[\begin{array}{c} F(\tau )\\ Y(\tau ) \end{array}\right]d\tau \\ -\int_{t-T}^{t}\left(\left(\frac{\partial {\tilde{\Sigma }}_{h}}{\partial x}+{\tilde{\Phi }}_{h}\right)\left(L,\tau -t+T\right)Q_{0}^{-1}U\left(\tau \right)\right)d\tau \;. \end{array}$$

Note that in both cases, $M_{L}^{2},M_{L}^{1},M_{0}^{2}$, and $M_{0}^{1}$ can be expressed in known terms from the problem statement and also with the modulating functions obtained from the auxiliary models. Also note that (25) and (26) add a boundary condition to the auxiliary systems.

**Figure 1:**
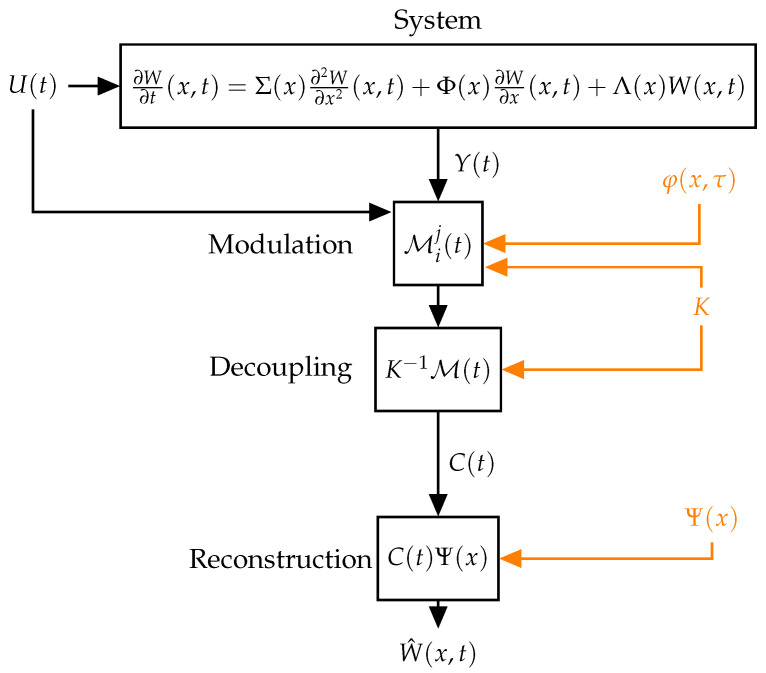
Diagram of the online implementation. Values calculated offline are in orange color.

For implementing the method, the procedure can be divided into two parts: offline and online, because there are some steps in the procedure that do not need to be iterated as Figure 1 demonstrates. The procedure can be resumed in the following steps:

**Offline part**

1. Define parameters for orthonormal basis calculation: approximation order *N*, function basis, weight function v(x);
2. Gram–Schmidt procedure to obtain the orthonormal basis $\Psi \left(x\right)=[\psi_{0}\left(x\right),\ldots ,$$\psi_{N}\left(x\right)]$;
3. Create and solve auxiliary models (14) with initial condition v(x)Ψ(x) and control scheme η to achieve ξ(x,T)=0. The solution is ξ(x,σ);
4. Inverse time transformation (17) to obtain modulating functions φ(x,τ).

**Online part**

1. Measurement in the system;
2. Calculation of the modulation kernels with (18);
3. Decoupling of the coefficients with (19);
4. Calculation of the states with (20).

The heaviest computational part of the whole process is the solution of the auxiliary models since it is a coupled PDE solution. However, it can be done offline. In the online part, the decoupling and calculation of the states are matrix multiplications without further complications. Also the matrices $K^{-1}$ and Ψ(x) can be calculated offline, with no need for actualization in the online section. The modulation part implies a numerical integration that is a heavier computational part to do online. This integration is done with numerical methods such as the trapezoidal rule or Newton–Cotes formulas for further improvement.

## 4. Real-Time Implementation and Simulation Results

The method presented in the last section provides a solution to the state estimation problem. In this section, an implementation is presented in order to exemplify the capabilities and the lower computational cost of the method. Simulations show the performance of the observer and a comparison with the conventional backstepping observer.

### 4.1. Example Problem Setup

In order to exemplify the use and efficiency of the method, a linearized chemical tubular reactor model is used for the simulation. The coupled temperature-concentration system of chemical tubular reactors is given by [25]. Linearization about the steady state and taking the average values of the coefficients yields

(27) $$\left\{\begin{array}{cc} \frac{\partial W}{\partial t}\; & =\Sigma \frac{\partial^{2}W}{\partial x^{2}}+\Lambda W\\ \Sigma & =\left[\begin{array}{cc} 0.14 & 0\\ 0 & 0.16 \end{array}\right],\Lambda =\left[\begin{array}{cc} -0.065 & -0.146\\ -0.130 & -0.293 \end{array}\right]\\ W(x,t) & ={\left[\begin{array}{cc} w_{1}\left(x,t\right) & w_{2}\left(x,t\right) \end{array}\right]}^{⊤} \end{array}\right.$$

with Neumann boundary condition

(28) $$\frac{\partial W}{\partial x}\left(0,t\right)=0,$$

known actuation at the boundary

(29) W(L,t)=U(t)

and measurement at the boundary

(30) $$Y\left(t\right)=\frac{\partial W}{\partial x}\left(L,t\right).$$

The problem is to estimate the distributed state W(x,t) based on the knowledge of the actuation U(t) and the measurement Y(t).

### 4.2. Solution of the Problem

For the solution of the problem the same argument explored in Section 3 is used, but with $x^{*}=L$. In this case, the auxiliary systems are similar to (14) and read as follows:

(31) $$\left\{\begin{array}{cc} \frac{\partial \xi^{m}}{\partial \sigma }\left(x,\sigma \right) & =\bar{\Sigma }\frac{\partial^{2}\xi^{m}}{\partial x^{2}}\left(x,\sigma \right)+\bar{\Lambda }\xi^{m}\left(x,\sigma \right)\\ \bar{\Sigma } & =\left[\begin{array}{cc} \frac{k_{1}}{k_{1}}\epsilon_{11} & \frac{k_{2}}{k_{1}}\epsilon_{21}\\ \frac{k_{1}}{k_{2}}\epsilon_{21} & \frac{k_{2}}{k_{2}}\epsilon_{22} \end{array}\right]=\left[\begin{array}{cc} \epsilon_{11} & 0\\ 0 & \epsilon_{22} \end{array}\right]\\ \bar{\Lambda } & =\left[\begin{array}{cc} \lambda_{11} & \frac{k_{2}}{k_{1}}\lambda_{12}\\ \frac{k_{1}}{k_{2}}\lambda_{21} & \lambda_{22} \end{array}\right]\\ \xi^{m}\left(x,0\right) & =v\left(x\right)\psi^{m}\left(x\right)\\ \xi^{m}\left(x,T\right) & =0\\ \xi^{m}\left(0,\sigma \right) & ={\tilde{\eta }}^{m}\left(\sigma \right). \end{array}\right.$$

For the present problem with $x^{*}=L$ the modulation kernel can be reformulated as

$$\begin{array}{c} \sum_{i,j=1}^{n}k_{hi}\int_{t-T}^{t}\left(M_{L}^{2}+M_{0}^{2}\right)\left[\epsilon_{ij}\left(x\right)\varphi_{hi}^{m},w_{j}\right]+\left(M_{L}^{1}+M_{0}^{1}\right)\left[\phi_{ij}\left(x\right)\varphi_{hi}^{m},w_{j}\right]d\tau \\ =\int_{t-T}^{t}\left[\begin{array}{cc} -{\tilde{\Sigma }}_{h}\left(0,\tau -t+T\right) & \frac{\partial {\tilde{\Sigma }}_{h}}{\partial x}\left(0,\tau -t+T\right) \end{array}\right]\left[\begin{array}{c} \frac{\partial W}{\partial x}\left(0,\tau \right)\\ W(0,\tau ) \end{array}\right]d\tau \\ +\int_{t-T}^{t}\left[\begin{array}{cc} {\tilde{\Sigma }}_{h}\left(L,\tau -t+T\right) & -\frac{\partial {\tilde{\Sigma }}_{h}}{\partial x}\left(L,\tau -t+T\right) \end{array}\right]\left[\begin{array}{c} \frac{\partial W}{\partial x}\left(L,\tau \right)\\ W(L,\tau ) \end{array}\right]d\tau \end{array}$$

and using the problem conditions from (2), (28) and (30) we get

$$\begin{array}{c} \sum_{i,j=1}^{n}k_{hi}\int_{t-T}^{t}\left(M_{L}^{2}+M_{0}^{2}\right)\left[\epsilon_{ij}\left(x\right)\varphi_{hi}^{m},w_{j}\right]d\tau \\ =\int_{t-T}^{t}\left[\begin{array}{cc} -{\tilde{\Sigma }}_{h}\left(0,\tau -t+T\right) & \frac{\partial {\tilde{\Sigma }}_{h}}{\partial x}\left(0,\tau -t+T\right) \end{array}\right]\left[\begin{array}{c} 0\\ W(0,\tau ) \end{array}\right]d\tau \\ +\int_{t-T}^{t}\left[\begin{array}{cc} {\tilde{\Sigma }}_{h}\left(L,\tau -t+T\right) & -\frac{\partial {\tilde{\Sigma }}_{h}}{\partial x}\left(L,\tau -t+T\right) \end{array}\right]\left[\begin{array}{c} Y(\tau )\\ U(\tau ) \end{array}\right]d\tau .\; \end{array}$$

Imposing on the modulating functions the boundary conditions

(32) $$\frac{\partial \varphi_{hi}^{m}}{\partial x}\left(0,\tau \right)=0,\;\;1\leq h,i\leq 2,$$

then $\frac{\partial {\tilde{\Sigma }}_{h}}{\partial x}=0$ and consequently the modulation kernel can be reduced to

$$\begin{array}{c} \sum_{i,j=1}^{n}k_{hi}\int_{t-T}^{t}\left(M_{L}^{2}+M_{0}^{2}\right)\left[\epsilon_{ij}\left(x\right)\varphi_{hi}^{m},w_{j}\right]d\tau =\int_{t-T}^{t}\left[\begin{array}{cc} {\tilde{\Sigma }}_{h}\left(L,\tau -t+T\right) & -\frac{\partial {\tilde{\Sigma }}_{h}}{\partial x}\left(L,\tau -t+T\right) \end{array}\right]\left[\begin{array}{c} Y(\tau )\\ U(\tau ) \end{array}\right]d\tau \;. \end{array}$$

Adding the boundary condition of (32), the auxiliary system in (31) becomes

(33) $$\left\{\begin{array}{cc} \frac{\partial \xi^{m}}{\partial \sigma }\left(x,\sigma \right) & =\bar{\Sigma }\frac{\partial^{2}\xi^{m}}{\partial x^{2}}\left(x,\sigma \right)+\bar{\Lambda }\xi^{m}\left(x,\sigma \right)\\ \bar{\Sigma } & =\left[\begin{array}{cc} \epsilon_{11} & 0\\ 0 & \epsilon_{22} \end{array}\right],\bar{\Lambda }=\left[\begin{array}{cc} \lambda_{11} & \frac{k_{2}}{k_{1}}\lambda_{12}\\ \frac{k_{1}}{k_{2}}\lambda_{21} & \lambda_{22} \end{array}\right]\\ \xi^{m}\left(x,0\right) & =v\left(x\right)\psi^{m}\left(x\right)\\ \xi_{x}^{m}\left(0,\sigma \right) & =0\\ \xi^{m}\left(L,\sigma \right) & ={\tilde{\eta }}^{m}\left(\sigma \right)\\ \xi^{m}\left(x,T\right) & =0. \end{array}\right.$$

Meeting the conditions in (33) and applying the backwards time transformation (17), we obtain the modulating functions $\varphi_{hi}^{m}$, and thus ${\tilde{\Sigma }}_{h}$ and $\frac{\partial {\tilde{\Sigma }}_{h}}{\partial x}$. Finally, with

$$\begin{array}{cc} M_{h}^{m}\left(t\right)=\; & \int_{t-T}^{t}\left[\begin{array}{cc} {\tilde{\Sigma }}_{h}^{m}\left(L,\tau -t+T\right) & -{\frac{\partial {\tilde{\Sigma }}_{h}}{\partial x}}^{m}\left(L,\tau -t+T\right) \end{array}\right]\left[\begin{array}{c} Y(\tau )\\ U(\tau ) \end{array}\right]d\tau \end{array}$$

the reconstruction is possible along

$$W\left(x,t\right)\approx C\left(t\right)\Psi \left(x\right)=K^{-1}\left[\begin{array}{ccc} M_{1}^{0}\left(t\right) & \cdots & M_{1}^{N}\left(t\right)\\ M_{2}^{0}\left(t\right) & \cdots & M_{2}^{N}\left(t\right) \end{array}\right]\Psi \left(x\right).$$

### 4.3. Simulations

With the solution of the problem, explained in the last subsection, a simulation of the problem with different scenarios regarding the boundary conditions and noise presence is explored and also compared to the observer presented in [26]. The programming and graphical representations have been developed in MATLAB^®^.

In the following, the simulations and plots for the system have been done using the following actuation and initial condition, similar to [26], in order to keep the comparison fair:

(34) $$\begin{array}{c} W\left(1,t\right)=U\left(t\right)=\left[\begin{array}{c} 5\sin(t)\\ 10\sin(2t) \end{array}\right]\\ W\left(x,0\right)=\left[\begin{array}{c} \sin(\pi x)+\sin(3\pi x)\\ \sin(\pi x)+\sin(3\pi x) \end{array}\right]. \end{array}$$

The canonical polynomial basis $\left\{1,x,\cdots ,x^{N}\right\}$ and weight function $v\left(x\right)=x{\left(L-x\right)}^{2}$ are used in the Gram–Schmidt procedure to obtain the orthonormal basis. Then, the terms $v\left(x\right)\psi_{m}\left(x\right)$ are calculated to create and solve the auxiliary models described in (14). For this purpose we use

$$\begin{array}{c} K=\left[\begin{array}{cc} 1 & 1\\ 1 & -1 \end{array}\right]. \end{array}$$

Note that another value of *K* can be chosen, but be invertible to render possible the decoupling in (19). An other choice that has to be made is the control ${\tilde{\eta }}_{m}\left(\sigma \right)$ from (14) in order to stabilize the system in the time window [0,T] and fulfill the condition ξ(x,T)=0. For this, a backstepping controller, similar to [26] is used.

In order to demonstrate the effect of this degree of freedom, three cases are considered: No control (called Orig), a more aggressive control (called Cont1), and a more conservative control (called Cont2). For this, the backstepping control is used with different $\tilde{k}$ values.

**Figure 2:**
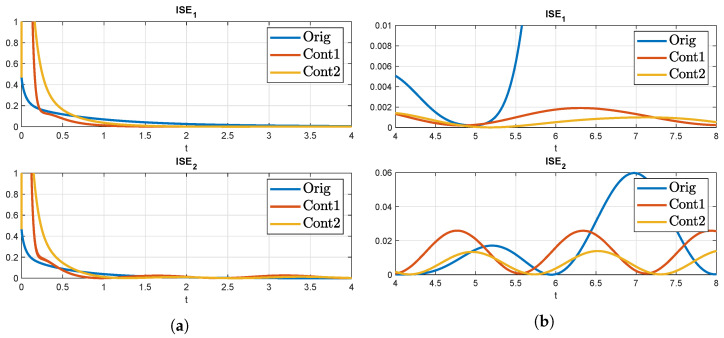
ISE with different control strategies for solving the MF on a stable coupled reaction-diffusion system. (**a**) Before *T*; (**b**) After *T*.

The first comparison to be explored is the use of different control strategies for the signal model control on the solution of the auxiliary systems from (14). In the presented example the three control strategies from the last subsection are used. For the simulation we have used a time grid resolution of $T_{s}=10^{-3}$ and a space grid resolution $X_{s}=2\times 10^{-2}$ with a time horizon length T=4. The results are illustrated in Figure 2.

**Figure 3:**
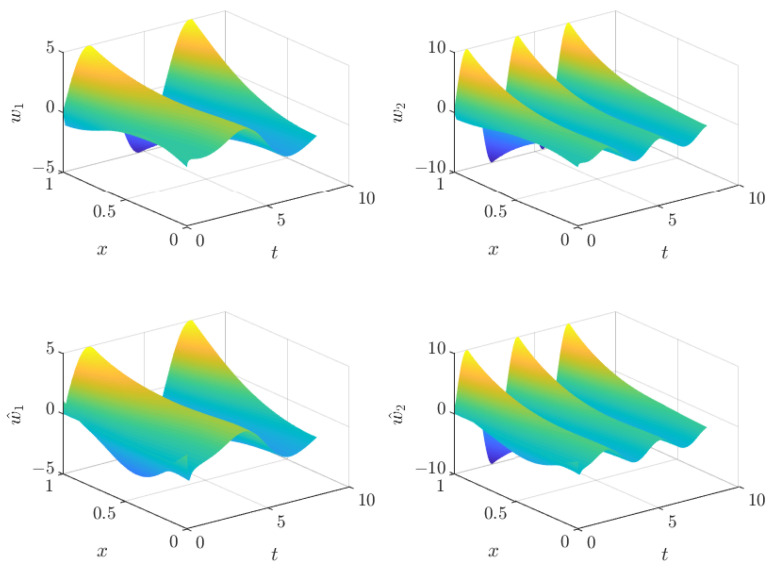
Comparison between real states (**top**) and estimated states (**bottom**).

The whole estimated state and the comparison with the real states can be seen in Figure 3. The estimated states are very similar, as confirmed by the ISE values.

**Figure 4:**
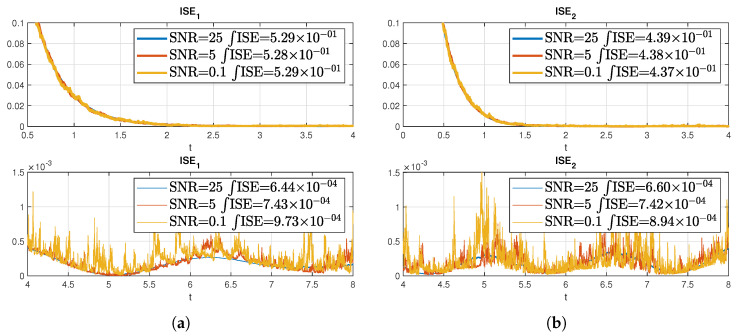
ISE with different SNRs on the measurement for the state estimation on a stable coupled reaction-diffusion system. (**a**) ISE of $w_{1}$ before and after *T*; (**b**) ISE of $w_{2}$ before and after *T*.

Another important factor to take into account is the effect of noise in the measurement during the state estimation. For this purpose, white noise with different SNRs was induced onto the measurement signal for the state estimation and the results are presented in Figure 4.

**Figure 5:**
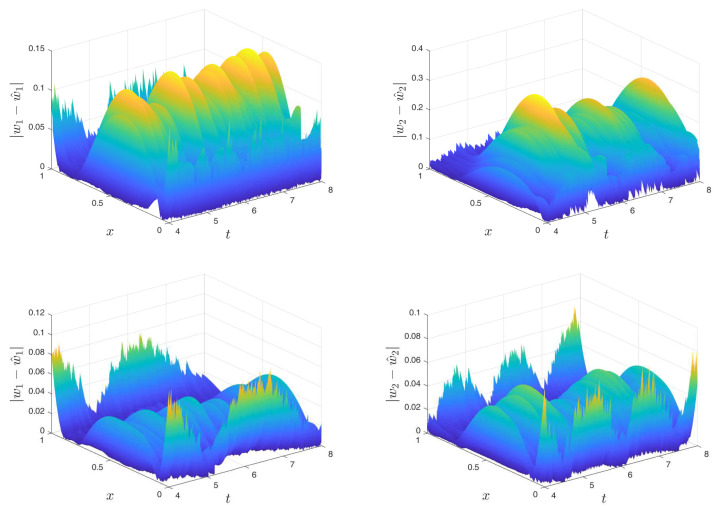
Absolute error for the estimation with SNR = 0.1 and $T_{s}=10^{-3}$ (**top**) and $T_{s}=10^{-4}$ (**bottom**) on a stable coupled reaction-diffusion system.

Naturally, the noise has an impact on the error as shown in the plots. This effect is also a deviation from the ISE, which with induced noise increases as the SNR increases. The effect of the noise is more visible in Figure 5 where the above figure is the absolute error for each state (sampling time of 1 × 10${}^{-3}$) and the row below is the absolute error (sampling time of 1 × 10${}^{-4}$) with an SNR of 0.1 in the measurement for each case. It can be seen that with increasing sampling time the error decreases in the middle of the space axis, while the error at the boundary practically does not. There also the noise impact is much clearer.

**Figure 6:**
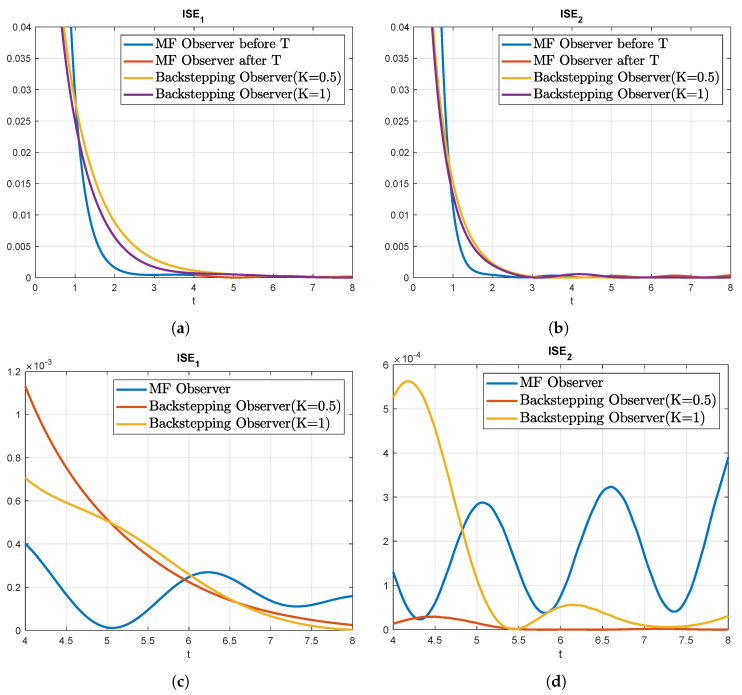
Comparison of ISE for the backstepping and MF observer in the state estimation on a stable coupled reaction-diffusion system. (**a**) ISE of $w_{1}$; (**b**) ISE of $w_{2}$; (**c**) ISE of $w_{1}$ after *T*; (**d**) ISE of $w_{2}$ after *T*.

Finally, under the same conditions the backstepping observer described in [26] is compared with the modulating function-based state estimation presented in this work. The same observer was applied as in [26]. For the sake of a fair comparison, the simulations are run with the same boundary conditions, that is (34). For the backstepping observer, the parameter $\tilde{k}$ has been set to $\tilde{k}=8$ according to [26], to $\tilde{k}=0.5$ according to Condition 1 in [26] and to $\tilde{k}=1$ according to Algorithm 2 in [26], in order to achieve a better performance of the observer. The MF observer uses a sampling time of $T_{s}=10^{-4}$, a time window of T=4, and the control strategy “Cont2” described before. The results of the comparison are shown in Figure 6.

In the comparison it can be observed that the MF observer converges after t=4 faster as can be seen in Table 1, with the ISE error for the first state and the second being smaller than the other observers. On the other hand, in the section before the time window, the backstepping observer keeps converging whereas the MF observer stops converging as it is by design. This is explained by the ISE error at t=8 for the MF observer being greater than the backstepping observers. The plot of the ISE error in Figure 6 shows this behavior and the influence of the values of the state at the boundary in the error of the MF observer after the time window *T*. This comparison shows the main differences between each approach and how the MF observer behaves with its non-asymptotic nature.

The results of the method in the unstable coupled reaction-diffusion system and with spatially varying coefficients are shown in Appendix A and the performance illustrates the application of the observer for these systems.

## 5. Conclusions

The presented modulating function-based observer achieves algebraic state estimation for linear coupled reaction-diffusion PDEs in finite time. Only boundary measurements as well as knowledge of the actuation signal is required. The modulating functions result from solving the corresponding auxiliary problems. They serve as kernels of the FIR filter realization for the reconstruction the modal state representation. The method constitutes a soft sensor for obtaining internal information on the coupled distributed processes, especially since measurements of the entire spatial domain are nearly impossible and the presented observer is capable of providing the state information with a boundary measurement. The algorithm has been implemented and its functionality is validated via simulation.

The observer approximates the original states based on a series expansion formulation of the PDE solution. In order to achieve this, the temporal coefficients of the respective basis expansion need to be calculated. In comparison to the original work [23], this calculation cannot be done straightforwardly by applying the modulation operator due to the coupling between states. To overcome this problem, a linear combination of every dynamic equation is constructed prior to the application of the modulation operator. The resulting auxiliary systems need to be solved offline for connecting the measurement information to the state representation. The auxiliary systems are adjoint to the original ones with modified coefficients related to the decoupling matrix *K* that is chosen appropriately. The modulating functionsare required to be zero at the end of the time window which is achieved by a signal model controller, transforming the problem into a stabilization task.

The central tuning parameters for the observer design are the sampling time $T_{s}$, used for the time integration and the sampling of the measured signals; the moving time horizon length *T*, adjusting the filtering properties of the algorithm; the approximation order *N*, that determines the accuracy of the state projection as well as the number of modulating functions.

There are three major sources of estimation errors. First, numerical errors are related to the accuracy of the numerical integration which depends on the sampling time $T_{s}$. It can be reduced using higher precision and thus, smaller values of $T_{s}$. Secondly, the projection error from the basis expansion approximation can be reduced by increasing the basis order and the grid resolution in *x*. Lastly, measurement noise affects the solution of the algebraic equations and is conversely amplified by higher projection orders as well as by a shorter horizon length *T*.

The method explained in the paper shows certain advantages with respect to other PDE observers such as the backstepping observers, as described in Table 2. This includes computationally lighter calculations for the state estimation since it only requires numerical integration and matrix multiplication in comparison to the backstepping observer that relies on the online solution of a coupled PDE for each time step. This advantage allows for an efficient real-time implementation of the modulating function-based method realized by FIR filter implementation as described in [23]. The robustness against sensor noise is demonstrated by the simulation results.

The main drawback of the presented approach is the sensitivity with respect to the sampling time affecting the performance of the observer. The simulative investigation shows that smaller values of the sampling time $T_{s}$ are crucial in order to achieve accurate results due to the numerical integration. This, however, raises the allocated memory size for storing past signal values and increases the number of processor operations. Another challenge is the state values dependency of the error since backstepping controllers with their convergence do not have this issue.

Next steps include the development of better signal model control methods for each auxiliary system in order to reduce the error of the estimated coefficients in relation to the moving time window length *T*. Furthermore, the influence and selection of the matrix *K* should be elaborated thoroughly in addition to the basic constraints established in this work. Another area of interest is the generation of even more algebraic equations in order to form an over-determined system of equations eventually leading to a higher robustness of the estimation procedure. Ultimately, an extension of the approach towards the inclusion of non-linear coupled systems could be explored as well as systems with time-varying coefficients in order to expand the methodology to a wider class of applicable systems.

## Figures and Tables

**Table 1:** ISE of $w_{1}$ at different times for the stable coupled reaction-diffusion PDE.

Observer	(t=2)	(t=4)	(t=8)
MF	$1.6\times 10^{-3}$	$4.0\times 10^{-4}$	$1.6\times 10^{-4}$
Backstepping $\left(\tilde{k}=1\right)$	$6.5\times 10^{-3}$	$7.1\times 10^{-4}$	$3.0\times 10^{-6}$
Backstepping $\left(\tilde{k}=0.5\right)$	$8.8\times 10^{-3}$	$1.1\times 10^{-3}$	$2.5\times 10^{-5}$

**Table 2:** Advantages, issues, inaccuracies, and future ideas for the method.

Advantages	Issues
Simple algebraic state estimator equations Good level of noise mitigation Real-time implementation	Dependency on sampling time Error depending on state values
**Inaccuracies**	**Future Ideas**
Basis expansion approximation Error at the boundaries	Advanced signal model control Consideration of non-linear terms

**Figure A1:**
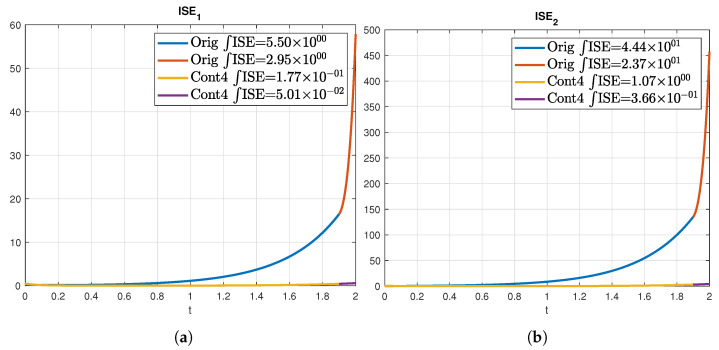
ISE with different control strategy for the MF solution for the state estimation on an unstable coupled reaction diffusion system. (**a**) ISE of $w_{1}$; (**b**) ISE of $w_{2}$.

**Figure A2:**
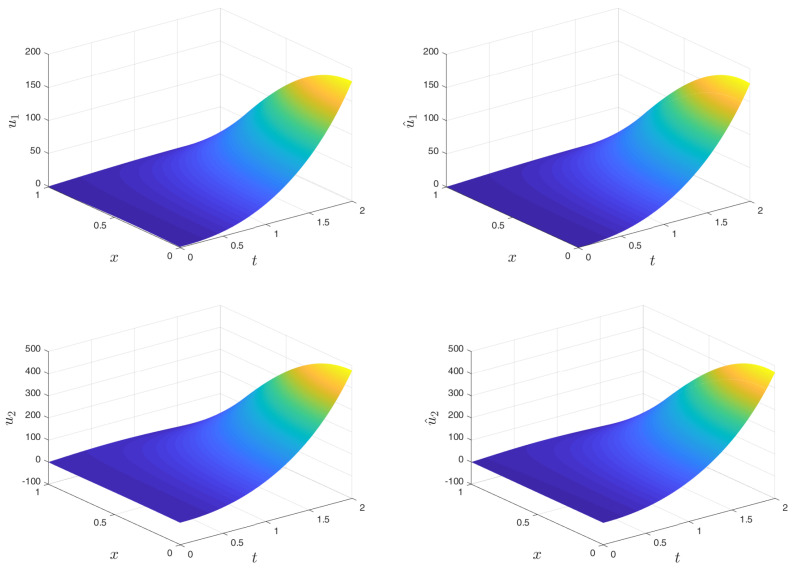
Original states (**top**) and estimated states (**bottom**) of an unstable coupled reaction diffusion system.

**Figure A3:**
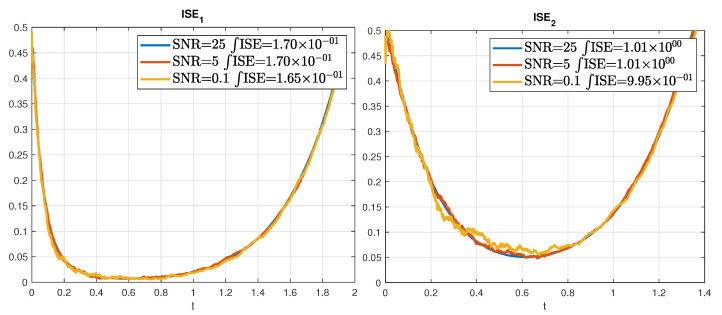
ISE for the state estimation on an unstable coupled reaction diffusion system.

**Figure A4:**
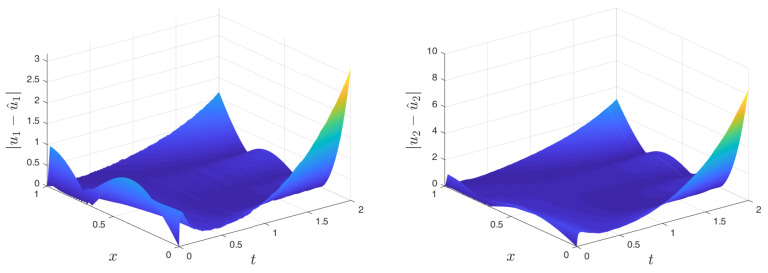
Comparison of the original and estimated state of an unstable coupled reaction diffusion system.

**Figure A5:**
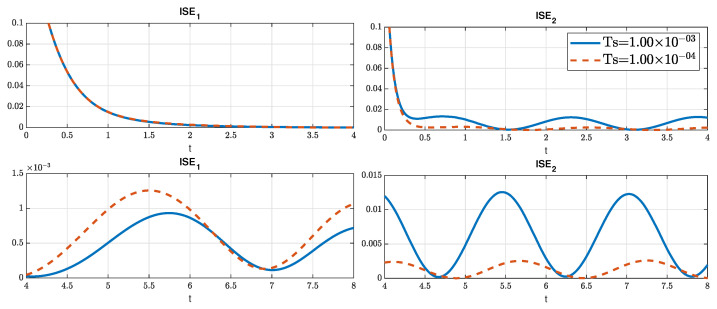
Effect of sampling time $T_{s}$ on the ISE for a coupled reaction diffusion system with spatially varying coefficients.

**Figure A6:**
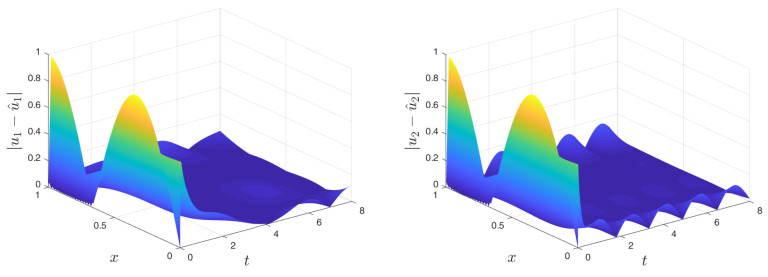
Absolute error of the estimation for each state on a coupled reaction diffusion system with spatially varying coefficients.

**Figure A7:**
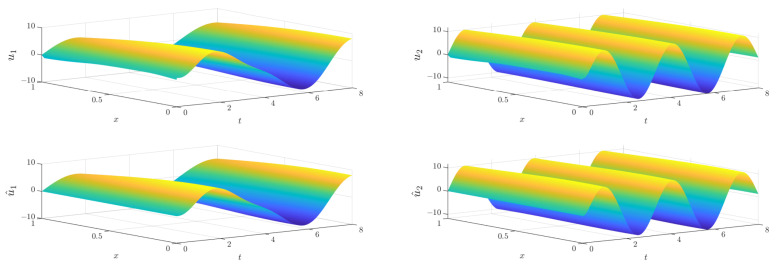
Comparison of the original and estimated state for the state estimation on a coupled reaction diffusion system with spatially varying coefficients.

**Figure A8:**
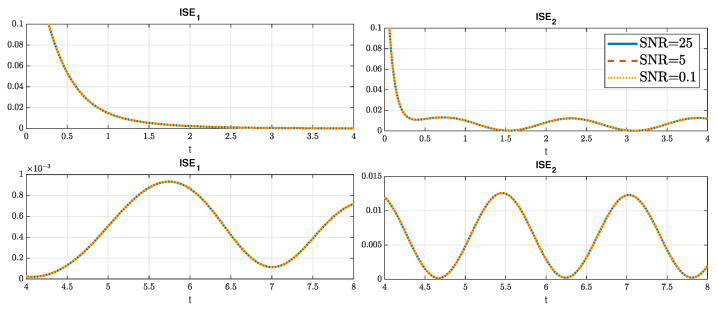
Effect of the noise on the ISE for each state on a coupled reaction diffusion system with spatially varying coefficients.

## Data Availability

Not applicable.

## Abbreviations

The following abbreviations are used in this manuscript:

PDE	Partial Differential Equation
ODE	Ordinary Differential Equation
MF	Modulating Function
SNR	Signal to Noise Ration
ISE	Integral Squared Error

## Appendix A

The following section presents more simulation results for the method in further scenarios and systems.

### Appendix A.1. Unstable Coupled Reaction-Diffusion PDE

The following linear 2-coupled reaction-diffusion PDE is used to demonstrate the performance of the MF observer in an unstable system:

(A1) $$\left\{\begin{array}{cc} \frac{\partial W}{\partial t}\; & =\Sigma \frac{\partial^{2}W}{\partial x^{2}}+\Lambda W\\ \Sigma & =\left[\begin{array}{cc} 2 & 0\\ 0 & 2 \end{array}\right],\Lambda =\left[\begin{array}{cc} 1 & 2\\ 4 & 5 \end{array}\right]\\ W(x,t) & ={\left[\begin{array}{cc} w_{1}\left(x,t\right) & w_{2}\left(x,t\right) \end{array}\right]}^{⊤}. \end{array}\right.$$

For the simulation, the same boundary conditions as for the former systems have been used. It is worth remarking that the system is unstable as can be noted in Figure A2, thus making necessary the use of a control strategy (Cont4) for the auxiliary system. This can be observed in Figure A1.

The error for the estimation without a control is much larger than the one with control for each state, which is due to the necessity to fulfill the condition ξ(x,T)=0. The results of the estimation can also be seen in Figure A2 where the original states are in the above figures and the estimated states in the lower figures, both showing similarity.

The effect of noise was also tested and the results are shown in Figure A3.

The absolute error is shown in Figure A4. The major error lies on the spatial boundaries, similar to the former system. It is also worth noticing that the error seems to increase as time increases since the state values also increase (see Figure A2). According to the error induced by the approximation of the condition ξ(x,T)=0, resulting from (9) and (10), an error is induced in the coefficient calculation and therefore an error in the state estimation. The distribution of the absolute error in the time and spatial axis can be seen in Figure A4.

### Appendix A.2. Coupled Reaction-Diffusion with Spatially Varying Coefficients PDE

The following linear 2-coupled reaction-diffusion PDE with spatially varying coefficients is used to demonstrate the performance of the MF observer in an unstable system:

(A2) $$\left\{\begin{array}{cc} \frac{\partial W}{\partial t}\; & =\Sigma \frac{\partial^{2}W}{\partial x^{2}}+\Lambda W\\ \Sigma & =\left[\begin{array}{cc} 1 & 0\\ 0 & 3 \end{array}\right],\Lambda =\left[\begin{array}{cc} 1 & x\\ x & 1 \end{array}\right]\\ W(x,t) & ={\left[\begin{array}{cc} w_{1}\left(x,t\right) & w_{2}\left(x,t\right) \end{array}\right]}^{⊤}. \end{array}\right.$$

The results with a sampling time of $1\times 10^{-3}$ and 1 × 10${}^{-4}$ are shown in Figure A5.

Performance of the observer also improves with a smaller sampling time such as the other systems, especially notable in the ISE of the second state.

The absolute error of the estimation is shown in Figure A6 and the comparison of the original and estimated state are shown in Figure A7.

The influence of noise shown in Figure A8, where the noise seems to have a smaller impact compared to the former systems, even increasing the SNR value as the plots indicate.

The appendix has shown the performance of the observer in further coupled reaction-diffusion PDEs and demonstrates how the MF observer can be applied in such cases with the different parameters.
